# Use of a Telemedicine Risk Assessment Tool to Predict the Risk of Hospitalization of 496 Outpatients With COVID-19: Retrospective Analysis

**DOI:** 10.2196/25075

**Published:** 2021-04-30

**Authors:** James B O'Keefe, Elizabeth J Tong, Thomas H Taylor Jr, Ghazala A Datoo O’Keefe, David C Tong

**Affiliations:** 1 Division of General Internal Medicine Department of Medicine Emory University School of Medicine Atlanta, GA United States; 2 Taylor Engineering, Inc Atlanta, GA United States; 3 Section of Vitreoretinal Surgery and Diseases, Section of Uveitis and Vasculitis Department of Ophthalmology Emory University School of Medicine Atlanta, GA United States; 4 Division of Hospital Medicine Department of Medicine Emory University School of Medicine Atlanta, GA United States

**Keywords:** COVID-19, SARS-CoV-2, nonhospitalized, risk assessment, outpatient, outcomes, telemedicine

## Abstract

**Background:**

Risk assessment of patients with acute COVID-19 in a telemedicine context is not well described. In settings of large numbers of patients, a risk assessment tool may guide resource allocation not only for patient care but also for maximum health care and public health benefit.

**Objective:**

The goal of this study was to determine whether a COVID-19 telemedicine risk assessment tool accurately predicts hospitalizations.

**Methods:**

We conducted a retrospective study of a COVID-19 telemedicine home monitoring program serving health care workers and the community in Atlanta, Georgia, with enrollment from March 24 to May 26, 2020; the final call range was from March 27 to June 19, 2020. All patients were assessed by medical providers using an institutional COVID-19 risk assessment tool designating patients as Tier 1 (low risk for hospitalization), Tier 2 (intermediate risk for hospitalization), or Tier 3 (high risk for hospitalization). Patients were followed with regular telephone calls to an endpoint of improvement or hospitalization. Using survival analysis by Cox regression with days to hospitalization as the metric, we analyzed the performance of the risk tiers and explored individual patient factors associated with risk of hospitalization.

**Results:**

Providers using the risk assessment rubric assigned 496 outpatients to tiers: Tier 1, 237 out of 496 (47.8%); Tier 2, 185 out of 496 (37.3%); and Tier 3, 74 out of 496 (14.9%). Subsequent hospitalizations numbered 3 out of 237 (1.3%) for Tier 1, 15 out of 185 (8.1%) for Tier 2, and 17 out of 74 (23%) for Tier 3. From a Cox regression model with age of 60 years or older, gender, and reported obesity as covariates, the adjusted hazard ratios for hospitalization using Tier 1 as reference were 3.74 (95% CI 1.06-13.27; *P*=.04) for Tier 2 and 10.87 (95% CI 3.09-38.27; *P*<.001) for Tier 3.

**Conclusions:**

A telemedicine risk assessment tool prospectively applied to an outpatient population with COVID-19 identified populations with low, intermediate, and high risk of hospitalization.

## Introduction

In March 2020, the identification of SARS-CoV-2 in the United States led to the rapid closure of elective medical care at many health care institutions, with redeployment of personnel to address the rising burden of COVID-19. In the US state of Georgia, the cumulative number of cases reported by the Department of Public Health rose from 84 cases on March 15, 2020, to 4231 cases by March 31, 2020.

It was recognized from early reports that the severity of COVID-19 varies from asymptomatic to life-threatening [1,2] and that most patients have mild illness and do not require hospitalization [3]. For these patients, the recommendation is to isolate at home and monitor symptoms under the care of a medical provider [4,5]. Many US medical centers have employed telemedicine and remote monitoring programs to provide this care [6-8]. Monitoring programs require investment and staffing [7]; it may be appropriate to focus these resources on those at highest risk of hospitalization for severe COVID-19. While it is recognized that certain groups (eg, older adults and patients with diabetes) have higher rates of hospitalization [9-12], there are no validated risk assessment tools that stratify risk for outpatients undergoing home monitoring [13]. The tools in existence often require in-person criteria (eg, vital signs, labs, and imaging) that are not available by telemedicine [13,14].

In order to better target care for outpatients with COVID-19, we created a risk assessment tool to assign patients a *risk tier* by incorporating age, comorbidities, symptom severity and course, and the ability to isolate—criteria highlighted in the initial US Centers for Disease Control and Prevention (CDC) guidance for home monitoring of patients with COVID-19 [4]. We prospectively applied this risk tool during the telemedicine assessment of outpatients recently diagnosed with COVID-19 in a home monitoring program. Patients were followed with regular phone calls until clinical improvement or hospitalization. In this retrospective study, we analyzed patient data gathered systematically at telemedicine intake visits, including patient characteristics and assigned risk tier, and used an outcome of hospitalization related to COVID-19. We hypothesized that the multifactorial tool would predict hospitalization rates.

## Methods

### Ethical Approval and Consent

The study was approved by the Emory University Institutional Review Board, which granted a waiver of consent and a waiver of Health Insurance Portability and Accountability Act authorization. The study was carried out in accordance with the principles embodied in the Declaration of Helsinki.

### Study Setting and Population

The study is a retrospective cohort investigation of outpatients with confirmed COVID-19 at Emory Healthcare, the largest academic health system in Georgia, serving the greater Atlanta metropolitan area. Testing was scheduled through a central COVID-19 hotline and performed at one screening clinic and one drive-through site, in addition to the emergency departments (EDs) at four hospitals. The test used was real-time reverse transcription–polymerase chain reaction (RT-PCR) detection of SARS-CoV-2 by nasopharyngeal swab. During the study period, testing for COVID-19 was available for symptomatic adults and prioritized (1) health care workers, (2) university students on campus, and (3) patients who were older or had medical comorbidities. Testing and monitoring of children (aged <18 years) was not available at Emory Healthcare. Patients with positive RT-PCR results were called by a dedicated result notification nurse team to provide self-care advice and refer for enrollment in the home monitoring program, named the Virtual Outpatient Monitoring Clinic (VOMC). Characteristics of the first 208 patients in VOMC [15] and the symptom course of VOMC patients [16] have been previously described.

The VOMC intake team included 14 physicians and 3 advanced practice providers (APPs) from two primary care clinics. VOMC follow-up call teams included 19 redeployed registered nurses (RNs) and 20 APPs. All intake providers were trained in the use of the risk assessment tool in a 1-hour webinar and conducted a median of 25 intake visits during the study period (IQR 36.5; range 5-99).

Enrollment criteria for this study included the following: (1) completion of new patient VOMC visit during the period of March 24 to May 26, 2020, and (2) documentation of positive RT-PCR result for SARS-CoV-2. Exclusion criteria included the following: (1) hospitalization prior to VOMC enrollment and (2) immediate discharge from VOMC—no follow-up calls—due to meeting CDC criteria for ending home isolation (≥14 days from symptom onset with resolution of fever and improvement in respiratory symptoms).

### Exposure

VOMC intake visits comprised a 40-minute nurse intake (ie, initial data entry) followed by a 40-minute physician or APP telemedicine visit including risk assessment. The clinical care pathway for outpatients with COVID-19 in the VOMC is outlined in Multimedia Appendix 1. The risk assessment tool used by the VOMC was created based on published data about risk factors for severe COVID-19 and the natural history of disease available in March 2020. Patients were assigned to a baseline *risk tier 1 to 3* by the provider upon completion of the VOMC intake visit that determined the planned frequency and duration of monitoring. Low-risk patients (Tier 1) received calls every other day for a minimum of 7 days from symptom onset. Intermediate-risk patients (Tier 2) received daily calls for a minimum of 14 days from symptom onset. High-risk patients (Tier 3) were called twice daily for a minimum of 21 days from symptom onset. There was no limit on duration of care, and calls would continue for all patients until symptom improvement or hospitalization, regardless of tier.

Details of the tier assignment by the VOMC risk assessment tool are in Multimedia Appendix 2. Tier 1 patients must meet *all* of the following criteria: aged <60 years; no comorbidities known to increase risk of severe COVID-19; no lower respiratory tract symptoms, except mild cough; and able to self-isolate. Tier 2 included patients aged 60 to 69 years without comorbidities and patients aged less than 60 years with moderate-risk comorbidities or with persistent symptoms (ie, no improvement) into the second week of illness. Tier 3 included patients meeting *any* of the following criteria: aged ≥70 years, younger age with specific high-risk comorbidity or multiple comorbidities, new or worsening lower respiratory symptoms, or uncertain ability to self-isolate. Providers were instructed to lower the risk tier by one level for patients whose intake visit occurred during the second week of illness if they reported improving symptoms, even if older age or comorbidities were present.

### Outcome

Hospitalization was the primary study outcome, consistent with the stated purpose of the risk assessment tool. ED visits and observation admissions were not included as events. Hospitalization at four Emory Healthcare acute care hospitals was determined by Emory Clinical Data Warehouse (CDW) queries, last performed on July 6, 2020. External hospitalizations were identified by chart review in (1) VOMC clinical notes, (2) administrative messages, and (3) hospitalization documentation in the Emory Healthcare electronic health record per data sharing agreements with other health systems. Loss to follow-up was minimal because VOMC patients were followed until symptom improvement and for specified minimum intervals—7, 14, and 21 days for Tiers 1, 2, and 3, respectively—whichever was longer.

### Covariates

Risk assessment data were obtained for all patients enrolling in the VOMC during an intake telemedicine visit utilizing synchronous two-way audio-video communication, with a telephone call as a backup option. VOMC providers completed a standard note template, including comorbidities (ie, past medical history and specific conditions with elevated COVID-19 severity risk), symptom description (ie, onset, severity, and course), social support and ability to isolate, and clinician-assigned risk tier using the risk assessment tool (Multimedia Appendix 2). These data were extracted from the completed VOMC intake notes by CDW query. Missing data were included by manual chart review by the authors (JO and GO) of provider free-text documentation in the intake note. Only data recorded at intake visits with initial risk tier were used; subsequent changes in illness severity and tier reassignments, based on worsening or improvement, were not used in the analysis. Actual age, BMI (calculated as weight in kilograms divided by height in meters squared), and race were obtained with a second CDW query of demographics and height and weight records. If a BMI of 30 or greater was recorded by the provider in the comorbidity portion the VOMC note, it was considered *reported obesity*. If a BMI of 30 or greater was identified by either the VOMC note or the inclusion of height and weight records, we considered this *corrected obesity* for analysis. As we observed underreporting of BMI values of 30 or greater in provider notes compared to height and weight records, we conducted sensitivity analyses to determine if preference for one metric or another substantially influenced results.

### Statistical Methods

Survival analysis was used to determine factors associated with hospitalization to evaluate the risk tier model. Initial unadjusted hazard ratios (HRs) were calculated using a Cox proportional hazards model. A multivariable model was then constructed using a Cox proportional hazards model. Time-varying covariates were identified by individual evaluation of covariates looking at Kaplan-Meier curves and testing for a statistically significant time-variable interaction. Covariates with *P* values less than .05 for the time interaction term were considered time-varying.

The models developed by backward and forward selection were then manually checked by adding and removing individual variables and assessing model fit. Cases with missing data were not included in analysis during the exploratory phase. The final model did not have any missing data. To provide odds ratios (ORs) for comparison, logistic regression was performed with the same variables as the Cox regression analysis. All analyses were conducted using SPSS statistical software, version 26 (IBM Corp).

### Proposed Simplified Tier Model

We considered covariates for a streamlined risk assessment model to simplify the tier-assignment process for more practical use.

## Results

### Participant Characteristics

We identified 551 patients completing a new VOMC visit from March 24, 2020, through May 26, 2020. We included 496 patients in the analysis after excluding 7 patients without a positive RT-PCR result, 25 patients hospitalized for COVID-19 prior to their VOMC visit, 2 patients sent to the ED and hospitalized at their first VOMC visit, 1 patient with a blank form, and 20 patients who met criteria for discharge—by duration of symptoms and improvement—and were, thus, not placed into a tier. During the study period—testing dates March 15 to May 22, 2020—the following number of nonhospitalized patients tested positive for SARS-CoV-2 by RT-PCR at Emory Healthcare: 730 in the outpatient setting and 170 in the ED. We do not have data on the patients who did not complete a VOMC intake visit.

The timing of the initial VOMC visit was similar between tiers (mean 9.3 days from symptom onset), and the mean follow-up was shorter for Tier 1 (mean 9.5 days, 95% CI 8.6-10.4) compared to the overall cohort (mean 13.1, 95% CI 12.2-13.9) (Table 1). The majority of the patients were female (330/496, 66.5%), 252 (50.8%) were Black, and 383 (77.2%) were under 60 years of age. Only 174 patients out of 496 (35.1%) reported no high-risk comorbidities, with hypertension (175/496, 35.3%) and reported BMI greater than 30 (147/496, 29.6%) as the most frequent comorbidities. Most patients (316/496, 63.7%) had mild symptoms or no symptoms at the time of the visit.

**Table 1:** Characteristics of outpatients with COVID-19 by assigned risk tier in a retrospective cohort from a telemedicine monitoring program in Atlanta, Georgia, with enrollment between March 24 and May 26, 2020.

Characteristic			All patients (N=496)		Tier 1^a^ (n=237)		Tier 2^a^ (n=185)		Tier 3^a^ (n=74)
Age (years), mean (95% CI)			47.6 (46.3-48.9)		41.5 (39.8-43.2)		52.5 (50.6-54.4)		54.9 (51.4-58.4)
Days from first symptom to intake visit, mean (95% CI)			9.3 (8.5-10.0)		8.9 (8.2-9.7)		10.0 (8.4-11.6)		8.4 (6.9-9.8)
Days from COVID-19 test to intake visit, mean (95% CI)			3.7 (3.4-3.9)		3.9 (3.4-4.4)		3.5 (3.0-3.9)		3.3 (2.7-4.0)
Follow-up duration (days from intake), mean (95% CI)			13.1 (12.2-13.9)		9.5 (8.6-10.4)		16.3 (14.8-17.7)		16.7 (14.0-19.3)
**Age category (years), n (%)**									
	18-29	78 (15.7)		65 (27.4)		10 (5.4)		3 (4.1)	
	30-39	84 (16.9)		49 (20.7)		26 (14.1)		9 (12.2)	
	40-49	106 (21.4)		50 (21.1)		39 (21.1)		17 (23.0)	
	50-59	115 (23.2)		48 (20.3)		50 (27.0)		17 (23.0)	
	60-69	84 (16.9)		21 (8.9)		45 (24.3)		18 (24.3)	
	≥70	29 (5.8)		4 (1.7)		15 (8.1)		10 (13.5)	
**Race, n (%)**									
	Black	252 (50.8)		109 (46.0)		102 (55.1)		41 (55.4)	
	White	97 (19.6)		47 (19.8)		36 (19.5)		14 (18.9)	
	Other	147 (29.6)		81 (34.2)		47 (25.4)		19 (25.7)	
**Gender, n (%)**									
	Female	330 (66.5)		156 (65.8)		125 (67.6)		49 (66.2)	
	Male	166 (33.5)		81 (34.2)		60 (32.4)		25 (33.8)	
**Comorbidities, n (%)**									
	Asthma	73 (14.7)		18 (7.6)		37 (20.0)		18 (24.3)	
	Cancer or malignancy	37 (7.5)		9 (3.8)		21 (11.4)		7 (9.5)	
	Chronic obstructive pulmonary disease	5 (1.0)		0 (0)		4 (2.2)		1 (1.4)	
	Coronary artery disease	24 (4.8)		1 (0.4)		11 (5.9)		12 (16.2)	
	Diabetes	69 (13.9)		10 (4.2)		35 (18.9)		24 (32.4)	
	Drug abuse or addiction	4 (0.8)		0 (0)		4 (2.2)		0 (0)	
	Heart failure	10 (2.0)		2 (0.8)		4 (2.2)		4 (5.4)	
	Hypertension	175 (35.3)		42 (17.7)		91 (49.2)		42 (56.8)	
	Immune suppression	30 (6.0)		9 (3.8)		11 (5.9)		10 (13.5)	
	Lung disease	17 (3.4)		3 (1.3)		9 (4.9)		5 (6.8)	
	Reported obesity^b^	147 (29.6)		52 (21.9)		63 (34.1)		32 (43.2)	
	Corrected obesity^c^	212 (42.7)		87 (36.7)		85 (45.9)		40 (54.1)	
	Renal disease	16 (3.2)		3 (1.3)		6 (3.2)		7 (9.5)	
**Number of diagnoses, n (%)**									
	0 (healthy)	174 (35.1)		129 (54.4)		35 (18.9)		10 (13.5)	
	1	158 (31.9)		81 (34.2)		65 (35.1)		12 (16.2)	
	2	91 (18.3)		19 (8.0)		48 (25.9)		24 (32.4)	
	≥3	73 (14.7)		8 (3.4)		37 (20.0)		28 (37.8)	
**Ability to self-isolate safely, n (%)**									
	Adequate	409 (82.5)		205 (86.5)		156 (84.3)		48 (64.9)	
	Inadequate	9 (1.8)		1 (0.4)		3 (1.6)		5 (6.8)	
	Unknown	78 (15.7)		31 (13.1)		26 (14.1)		21 (28.4)	
**Severity of symptoms, n (%)**									
	None or mild	316 (63.7)		204 (86.1)		102 (55.1)		10 (13.5)	
	Moderate	134 (27.0)		18 (7.6)		69 (37.3)		47 (63.5)	
	Severe	9 (1.8)		0 (0)		0 (0)		9 (12.2)	
	Unknown	37 (7.5)		15 (6.3)		14 (7.6)		8 (10.8)	
**Symptoms course, n (%)**									
	Improving	264 (53.2)		156 (65.8)		90 (48.6)		18 (24.3)	
	Stable	155 (31.3)		61 (25.7)		65 (35.1)		29 (39.2)	
	Worsening	31 (6.3)		0 (0)		14 (7.6)		17 (23.0)	
	Unknown	46 (9.3)		20 (8.4)		16 (8.6)		10 (13.5)	

^a^Risk tiers: Tier 1 = low risk, Tier 2 = intermediate risk, and Tier 3 = high risk (Multimedia Appendix 2).

^b^BMI ≥30 recorded in Virtual Outpatient Monitoring Clinic (VOMC) intake note by provider; BMI is calculated as weight in kilograms divided by height in meters squared.

^c^BMI ≥30 determined by height and weight data in electronic medical record or recorded in VOMC intake note; BMI is calculated as weight in kilograms divided by height in meters squared.

### Univariate Analysis

We identified 35 VOMC patients requiring hospitalization and 461 patients who did not require hospitalization during the follow-up period. There were no deaths during VOMC care at home; 2 patients died during hospitalization and a third died shortly after hospitalization while in hospice care. Statistically significant factors for hospitalization included risk tier, age, coronary artery disease, diabetes mellitus, heart failure, reported obesity (BMI ≥30), two comorbidities, three or more comorbidities, severe symptom rating, and worsening symptom course (Table 2). Of the patients initially categorized as Tier 3, 17 out of 74 (23%) were hospitalized in the course of their care, compared with 15 out of 185 (8.1%) Tier 2 patients and 3 out of 237 (1.3%) Tier 1 patients. Among 35 hospitalized patients, the median days to admission from symptom onset was 8 in Tier 3, 11 in Tier 2, and 13 in Tier 1. Tier level had the highest unadjusted HR of all factors, with 5.29 for Tier 2 and 16.24 for Tier 3 in comparison to Tier 1.

**Table 2:** Characteristics of patients by outcome of hospitalization in a retrospective cohort from a telemedicine monitoring program in Atlanta, Georgia, with enrollment between March 24 and May 26, 2020.

Characteristic			Nonhospitalized patients (n=461)		Hospitalized patients (n=35)		Unadjusted hazard ratio (95% CI)		*P* value
Age (years), mean (95% CI)			46.7 (45.4-48.1)		59.1 (55.2-63.1)		N/A^a^		<.001^b^
Days from first symptom to visit, mean (95% CI)			9.4 (8.6-10.2)		7.4 (5.2-9.6)		N/A		.16^b^
Days from COVID-19 test to visit, mean (95% CI)			3.7 (3.4-4.0)		2.7 (1.9-3.5)		N/A		.09^b^
Follow-up duration (days), mean (95% CI)			13.4 (12.6-14.3)		8.5 (5.0-12.1)		N/A		.003^b^
**Age category (years), n (%)**									
	18-29 (n=78)	78 (16.9)		0 (0)		N/A		—^c^	
	30-39 (n=84)	81 (17.6)		3 (8.6)		Reference		—	
	40-49 (n=106)	103 (22.3)		3 (8.6)		0.71 (0.14-3.53)		.68	
	50-59 (n=115)	105 (22.8)		10 (28.6)		2.16 (0.59-7.85)		.24	
	60-69 (n=84)	68 (14.8)		16 (45.7)		4.89 (1.42-16.79)		.01	
	≥70 (n=29)	26 (5.6)		3 (8.6)		2.32 (0.47-11.52)		.31	
**Race, n (%)**									
	Other (n=147)	141 (30.6)		6 (17.1)		Reference		—	
	Black (n=252)	234 (50.8)		18 (51.4)		1.59 (0.63-4.01)		.33	
	White (n=97)	86 (18.7)		11 (31.4)		2.59 (0.96-7.01)		.06	
**Gender, n (%)**									
	Female (n=330)	311 (67.5)		19 (54.3)		Reference		—	
	Male (n=166)	150 (32.5)		16 (45.7)		1.76 (0.91-3.43)		.10	
**Comorbidities, n (%)**									
	Asthma (n=73)	67 (14.5)		6 (17.1)		1.07 (0.44-2.59)		.88	
	Cancer or malignancy (n=37)	34 (7.4)		3 (8.6)		1.07 (0.33-3.50)		.91	
	Chronic obstructive pulmonary disease (n=5)	4 (0.9)		1 (2.9)		2.58 (0.35-18.84)		.35	
	Coronary artery disease (n=24)	18 (3.9)		6 (17.1)		3.71 (1.54-8.96)		.004	
	Diabetes (n=69)	56 (12.1)		13 (37.1)		3.59 (1.81-7.12)		<.001	
	Drug abuse or addiction (n=4)	3 (0.7)		1 (2.9)		4.29 (0.59-31.45)		.15	
	Heart failure (n=10)	6 (1.3)		4 (11.4)		5.84 (2.06-16.55)		<.001	
	Hypertension (n=175)	157 (34.1)		18 (51.4)		1.75 (0.90-3.40)		.10	
	Immune suppression (n=30)	30 (6.5)		0 (0)		0.05 (0.00-16.22)		.30	
	Lung disease (n=17)	14 (3.0)		3 (8.6)		2.10 (0.64-6.88)		.22	
	Obesity reported^d^ (n=147)	130 (28.2)		17 (48.6)		2.27 (1.17-4.41)		.02	
	Obesity corrected^e^ (n=212)	186 (87.7)		26 (74.3)		3.83 (1.80-8.18)		<.001	
	Renal disease (n=16)	13 (2.8)		3 (8.6)		2.35 (0.72-7.71)		.16	
**Number of diagnoses, n (%)**									
	0 (healthy) (n=174)	170 (36.9)		4 (11.4)		Reference		—	
	1 (n=158)	148 (32.1)		10 (28.6)		2.61 (0.82-8.34)		.11	
	2 (n=91)	83 (18.0)		8 (22.9)		3.43 (1.03-11.40)		.04	
	≥3 (n=73)	60 (13.0)		13 (37.1)		6.77 (2.20-20.83)		<.001	
**Ability to self-isolate safely, n (%)**									
	Adequate (n=409)	383 (83.1)		26 (74.3)		Reference		—	
	Inadequate (n=9)	7 (1.5)		2 (5.7)		3.80 (0.90-16.05)		.07	
	Unknown (n=78)	71 (15.4)		7 (20.0)		Unknown		—	
**Severity of symptoms, n (%)**									
	None or mild (n=316)	301 (65.3)		15 (42.9)		Reference		—	
	Moderate (n=134)	121 (26.2)		13 (37.1)		1.79 (0.85-3.77)		.13	
	Severe (n=9)	6 (1.3)		3 (8.6)		6.82 (1.95-23.83)		.003	
	Unknown (n=37)	33 (7.2)		4 (11.4)		Unknown		—	
**Symptoms course, n (%)**									
	Improving (n=264)	253 (54.9)		11 (31.4)		Reference		—	
	Stable (n=155)	143 (31.0)		12 (34.3)		1.84 (0.81-4.17)		.15	
	Worsening (n=31)	24 (5.2)		7 (20.0)		5.43 (2.10-14.03)		<.001	
	Unknown (n=46)	41 (8.9)		5 (14.3)		Unknown		—	
**Tier, n (%)**									
	1 (n=237)	234 (50.8)		3 (8.6)		Reference		—	
	2 (n=185)	170 (36.9)		15 (42.9)		5.29 (1.53-18.32)		.009	
	3 (n=74)	57 (12.4)		17 (48.6)		16.24 (4.74-55.59)		<.001	

^a^N/A: not applicable; unadjusted hazard ratio was not calculated.

^b^*P* value was based on a *t* test.

^c^Not calculated, either because the unadjusted hazard ratio value was not calculated or was unknown or because the characteristic was used as reference.

^d^BMI ≥30 recorded in Virtual Outpatient Monitoring Clinic (VOMC) intake note by provider; BMI is calculated as weight in kilograms divided by height in meters squared.

^e^BMI ≥30 determined by height and weight data in electronic medical record or recorded in VOMC intake note; BMI is calculated as weight in kilograms divided by height in meters squared.

### Multivariable Analysis

**Figure 1:**
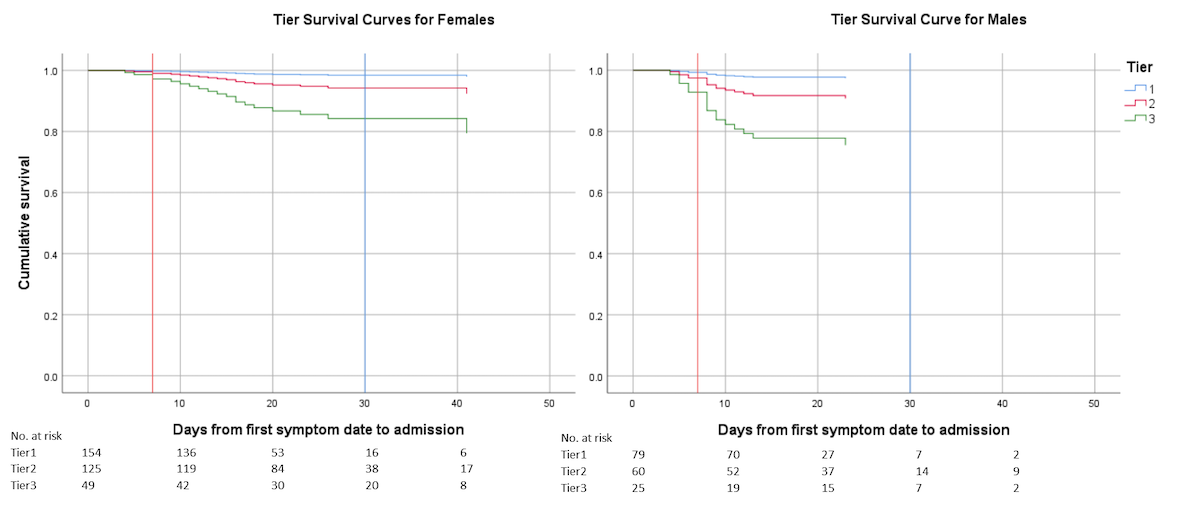
Cox regression survival curves for hospitalization by risk tier in the outpatient telemedicine cohort.

The final model that predicts hospitalization among outpatients in VOMC includes (1) risk tier, (2) reported obesity, (3) aged ≥60 years, and (4) gender as strata (Table 3). This model had an overall fit that was statistically significant (*P*<.001). Covariates other than gender satisfied the proportional hazards assumption. Even though the risk tier rubric does take into account both age and obesity, both of these covariates remained statistically significant with HRs greater than 2 and so were retained in the final model (Multimedia Appendix 3). Gender was found to be a time-varying covariate (Multimedia Appendix 3) and was, therefore, analyzed by stratum [17]. The adjusted HRs for Tiers 2 and 3 compared to Tier 1 were 3.74 (95% CI 1.06-13.27; *P*=.04) and 10.87 (95% CI 3.09-38.27; *P*<.001), respectively. Age of 60 years or older had an adjusted HR of 2.53 (95% CI 1.27-5.02; *P*=.008) and reported obesity had an adjusted HR of 2.09 (95% CI 1.06-4.13; *P*=.03). Survival curves (Figure 1) show days from symptom onset to hospitalization by tier. Males were hospitalized earlier and more often than females. Logistic regression performed with the same variables to shadow the Cox regression analysis found similar results with adjusted ORs of 4.87 for Tier 2 and 15.38 for Tier 3 compared to Tier 1. Age of 60 years or older and reported obesity both had adjusted ORs similar to their adjusted HRs (Table 3). Gender was not statistically significant but was kept in the logistic regression model for comparison to the survival analysis.

**Table 3:** Hazard ratios (HRs) and odds ratios (ORs) for variables with significant predictive value for hospitalization in the outpatient telemedicine cohort.

Variable	Unadjusted HR (95% CI)	*P* value	Adjusted HR^a^ (95% CI)	*P* value	Adjusted OR^b^ (95% CI)	*P* value
Tier 1	Reference	N/A^c^	Reference	N/A	Reference	N/A
Tier 2	5.29 (1.53-18.32)	.009	3.74 (1.06-13.27)	.04	4.87 (1.35-17.57)	.02
Tier 3	16.24 (4.74-55.59)	<.001	10.87 (3.09-38.27)	<.001	15.38 (4.21-56.20)	<.001
Aged ≥60 years	3.77 (1.94-7.34)	<.001	2.53 (1.27-5.02)	.008	2.94 (1.38-6.25)	.005
Reported obesity	2.27 (1.17-4.41)	.02	2.09 (1.06-4.13)	.03	2.17 (1.01-4.67)	.048
Male	1.76 (0.91-3.43)	.10	Analyzed by strata	N/A	1.94 (0.91-4.18)	.09

^a^Cox overall model of fit: χ^2^_4_=41.4; *P*<.001.

^b^Logistic regression overall model of fit: χ^2^_5_=50.8; *P*<.001.

^c^N/A: not applicable; *P* value was not calculated because the variable was used as the reference (in the case of Tier 1) or because the variable was analyzed by strata (in the case of gender).

### Sensitivity Analysis

We performed sensitivity analysis for obesity to see if using the actual BMI (ie, corrected obesity) would be more predictive than reported obesity from the VOMC note. The adjusted HR for corrected obesity was 3.783 (95% CI 1.761-8.126; *P*<.001) with only minor changes in the HR and *P* values for tier and for age of 60 years or older (Multimedia Appendix 4).

### Proposed Simplified Tier Model

We looked at factors associated with hospitalization to propose a streamlined risk assessment model to predict which patients in the VOMC setting will not require hospitalization during COVID-19 illness. Defining a new Tier 1 as age of less than 60 years, no high-risk comorbidities, able to self-isolate, symptom severity mild or none, and symptom course stable or improving, we find a model with no hospitalizations for proposed Tier 1 patients (Table 4).

**Table 4:** Proposed simplified risk assessment for *new Tier 1*^a^ low-risk patients tested in the study cohort.

Tier	Hospital admission, n (%)	
	No	Yes
1 (n=114)	114 (100)	0 (0)
2 and 3 (n=382)	347 (90.8)	35 (9.2)

^a^The proposed four-criteria model for *new Tier 1* is as follows: (1) aged <60 years, (2) no at-risk comorbidities, (3) symptoms mild and stable or improving, and (4) able to self-isolate.

### Data Sharing Statement

Deidentified data are available in a public, open access repository [18].

## Discussion

### Principal Findings

This study describes the outcomes of outpatients with confirmed COVID-19 who participated in a standardized telemedicine risk assessment and telephone monitoring program. We found that the *risk tiers* designated by a multifactorial tool predicts hospitalization rates more strongly than individual variables. We also found that age and obesity still remained significant predictors even though they were part of the risk assessment. It is likely that providers weighed initial symptom severity more in their assessment than either age or obesity, as initial symptom severity was not significant when risk tier was taken into account. Future iterations of the risk assessment tool should increase the relative weights of age and obesity. We note that many similar efforts to produce valid outpatient risk assessment tools are ongoing, but they have not yet been prospectively validated [13].

### Comparison to Previous Studies

The overall hospitalization rate in this outpatient cohort was 7%, which is lower than that in other populations reported in New York (51.9%) [11] and Louisiana (39.7%) [19], likely because testing in these cohorts was concentrated in EDs, with lower numbers of patients in the outpatient setting. A more comparable cohort of outpatients monitored by text messaging in Pennsylvania reported a low rate of ED use at approximately 7% but with limited follow-up for hospitalization [20]. The individual risk factors for hospitalization in this study are similar to those identified in earlier cohort studies, particularly age, male sex, and elevated BMI [11,12], but these studies did not include any multifactorial provider risk assessment rubric.

### Potential Applications

The identification of a small group of outpatients (ie, Tier 3) at the highest risk of hospitalization facilitates planning efforts for high-intensity outpatient monitoring with limited follow-up resources and may justify the expanded implementation of the risk assessment tool at the point of care. One question raised by a useful risk tier rubric is whether it can be codified into a computer-resident algorithm, an artificial intelligence (AI) application. We attempted models with tier as an output rather than input and using the objective and subjective notes and clinical observations as inputs. We were unable to develop such a model, evidently because the tier assignment includes several points where clinical judgment is required and applied. Incorporating that clinical judgment is necessary and is beyond our AI ability at this point.

The largest group identified by this risk assessment tool was Tier 1. These individuals were at low risk of hospitalization, with 3 admissions out of 237 patients. In order to rapidly identify individuals at low risk of hospitalization and, therefore, who require fewer monitoring resources, we were able to simplify criteria for a proposed *new Tier 1* four-item risk score. As additional remote monitoring tools become available (eg, automated text message surveys), this population may be appropriate to assign *as needed* follow-up instead of proactive monitoring calls.

### Strengths and Limitations

While the overall study design is retrospective, our program implemented the risk assessment tool prospectively for all new patients with COVID-19, and we were able to follow all patients until clinical improvement or hospitalization because of the availability of redeployed providers (ie, RNs and APPs), minimizing gaps in data. Furthermore, we found that the risk tiers tool predicted hospitalization risk with highly significant results in multivariate analysis and time-to-hospitalization survival analysis. This supports our hypothesis that inclusion of multiple factors in patient assessment (ie, age, risk factors, symptoms, and social factors) would most effectively identify absolute hospitalization risk and time to hospital admission.

A primary limitation of this single-center study is generalizability to other populations. We had a high proportion of working-age individuals in the first wave of the pandemic, and relatively few older adults and socially disadvantaged individuals are included in the study population. This may explain the lower hospitalization rate compared to cities with larger outbreaks. Furthermore, the time to enrollment in the VOMC (9.3 days) reflects the real-world practice at our clinic, but limits generalizability to settings (eg, urgent care) where patients may present earlier in the disease course. Another limitation is the existence of different levels of observation (ie, frequency of telephone calls, provider type for calls, and duration of follow-up calls) based on assigned tier, which may have impacted outcomes. We cannot speculate if and how more frequent calls would affect the likelihood of hospitalization. We also acknowledge the possibility of loss to follow-up: patients could end VOMC care on request and we do not have direct data for outside hospitalizations, although we reviewed all charts for documentation of such.

The risk assessment tool itself has limitations. First, it was not derived from an outpatient cohort, since none existed at the time, and was instead designed based on limited data available from reports of COVID-19 in hospitalized patients. Second, due to the differential risk posed by age ranges and specific comorbidities, the risk tool is relatively complex and required skilled medical providers to gather the necessary data. We trained a dedicated provider group in its use, but this limits external validity. Even in the optimal setting, we encountered underreporting issues (eg, reported obesity vs actual BMI).

### Future Directions

In subsequent waves of the pandemic, the majority of patients in our practice remain home during acute COVID-19. Telephone monitoring continues to provide care for high-risk patients without the ability to participate in automated programs. With the introduction of technologies such as wearable monitoring devices and advanced treatments (eg, monoclonal antibodies), the identification of high-risk patients who are most likely to benefit continues to be a high priority. In this context, the refinement and validation of risk assessment rubrics across clinical sites and in diverse populations remains important to COVID-19 outpatient care. Future investigation may consider rapid tools (eg, automated identification of highest and lowest risk groups at the time of presentation for testing) as well as validation of provider assessment tools such as ours within other centralized telemedicine programs.

### Conclusions

Our findings suggest that patients at low, intermediate, and high risk for hospitalization may be identified with a telemedicine risk assessment tool incorporating age, medical history, symptom severity, and social factors. The Tier 1 patients in our cohort had low hospitalization rates. We observed increasing odds of hospitalization in Tiers 2 and 3, respectively. External validation of these findings is necessary, but we also recognize that care delivery decisions need to be made immediately in the context of recently escalating cases in the COVID-19 pandemic. It is possible to use these data to create care models targeting the highest-risk patients during the highest-risk time periods, but further study of the safety and outcomes of this risk-based approach is needed. This study represents our initial experience with an outpatient telemedicine COVID-19 risk assessment tool. In the absence of clear guidelines on the risk stratification and duration of monitoring of outpatient COVID-19, these data may help guide resource allocation, planning of current care structures, and future research.

## Appendix

Multimedia Appendix 1 Clinical care pathway for Virtual Outpatient Management Clinic (VOMC).

Multimedia Appendix 2 COVID-19 telemedicine risk tier assessment tool.

Multimedia Appendix 3 Model building.

Multimedia Appendix 4 Sensitivity analysis for obesity.

## Abbreviations

AI: artificial intelligence
APP: advanced practice provider
CDC: US Centers for Disease Control and Prevention
CDW: Clinical Data Warehouse
ED: emergency department
HHS: US Department of Health and Human Services
HR: hazard ratio
HRSA: Health Resources and Services Administration
OR: odds ratio
RN: registered nurse
RT-PCR: reverse transcription–polymerase chain reaction
VOMC: Virtual Outpatient Monitoring Clinic
