# Supplementary material for: Use of a Telemedicine Risk Assessment Tool to Predict the Risk of Hospitalization of 496 Outpatients With COVID-19: Retrospective Analysis
Source: JMIR Public Health Surveill. 2021 Apr 30;7(4):e25075. doi: 10.2196/25075 (PMC8092025; doi:10.2196/25075)
Supplement: Multimedia Appendix 1 [file publichealth_v7i4e25075_app1.docx]

**Multimedia Appendix 1. Clinical care pathway for Virtual Outpatient Management Clinic (VOMC)**

**Outpatient COVID-19 testing criteria (March-April 2020):**

1. Symptomatic with either (a) fever, cough, or shortness of breath or (b) two symptoms from the following: sore throat, congestion, myalgias, fatigue, diarrhea, loss of smell.
2. Prioritize: (a) frontline healthcare workers, (b) students on-campus and health professions, (c) CDC employees, (d) established patients with risk factors (age, comorbidity, immunosuppression, work in a communal setting).

**VOMC enrollment criteria:**

1. COVID-19 diagnosis by nasopharyngeal PCR;
2. Outpatient (not hospitalized) at the time of PCR test result
3. Within isolation period (defined as the presence of symptoms related to COVID-19 currently or within 3 days OR within first 7 days of test result if asymptomatic)

**Intake:**

1. Initial visit by telemedicine (synchronous audio/video, reflex to telephone-only if technical difficulty);
2. Physician or advance practice provider (APP) completes intake template assessment including symptom history, past medical history, physical examination, and risk assessment (see box 2) then provides advice for (1) symptom management, (2) home isolation guidance, and (3) outpatient monitoring plan (see “follow-up” below).

**Follow-up (March-June 2020):**

1. Patients receive follow-up telephone calls on the following schedule:
   1. Low-risk (Tier 1) registered nurse (RN) call every other day for a minimum of 7 days from symptom onset AND 3 days after improvement in fevers (without antipyretics) and improvement in respiratory symptoms.
   2. Intermediate risk (Tier 2) RN call daily for a minimum of 14 days from symptom onset AND 3 days after improvement in fevers (without antipyretics) and improvement in respiratory symptoms.
   3. High risk (Tier 3) APP call twice daily for a minimum of 21 days* from symptom onset AND 3 days after improvement in fevers (without antipyretics) and improvement in respiratory symptoms.

*patient decreased to Tier 2 daily RN call for calls on days 15-21 if stable.

1. Patients with worsening symptoms could change tier after enrollment at provider discretion.
2. Patients with ongoing symptoms after the minimum interval may request additional calls to follow for symptom management.
3. Clinical concerns about symptom escalation or prolonged symptoms were addressed by VOMC providers, primary care physicians (PCPs), in-person care in an acute respiratory clinic unless the patient needed or chose to present to the emergency department (ED).
4. Patients could be followed by VOMC as appropriate for COVID-19 care after ED evaluation and/or hospital discharge.

**Disengagement and follow-up:**

1. At end of care, VOMC patients were given discharge instructions for monitoring and return to usual activity per national and local guidelines for end of isolation period.
2. PCPs (if available) were notified of VOMC intake and disengagement of VOMC care.
3. Quality survey: A subset of the initial patients (271 attempted, 158 reached) were called by VOMC RNs in May-June 2020 (at least 3 weeks after disengagement) to verify symptom resolution and verify number of ED or hospitalization visits related to COVID-19.
