# Supplementary material for: Use of a Telemedicine Risk Assessment Tool to Predict the Risk of Hospitalization of 496 Outpatients With COVID-19: Retrospective Analysis
Source: JMIR Public Health Surveill. 2021 Apr 30;7(4):e25075. doi: 10.2196/25075 (PMC8092025; doi:10.2196/25075)
Supplement: Multimedia Appendix 2 [file publichealth_v7i4e25075_app2.docx]

**Multimedia Appendix 2: COVID-19 Telemedicine Risk Tier Assessment Tool**

Version date: 4/5/2020
Authorship: James O’Keefe, M.D. (on behalf of the Paul W. Seavey Comprehensive Internal Medicine Clinic), Assistant Professor of Medicine, General Internal Medicine and Geriatrics, Emory University School of Medicine. Summary table courtesy Jason Higdon, MD.

**Purpose**:

1. Use telemedicine to assess patients with COVID-19 who are in isolation at home
2. Assign a level of risk (Risk Tier 1-3) for future hospitalization to guide monitoring plan

**Definition of “Risk Tier”:**

A Risk Tier summarizes our estimated risk for future hospitalization for a patient who is stable to monitor COVID-19 at the time of the assessment. This designation assigns the patient to a level of follow-up care (frequency of contact) within our Outpatient Virtual COVID-19 Management Clinic.

We determine a risk tier based on clinical assessment inclusive of patient-specific characteristics, current illness severity, recent clinical course, and social factors necessary for home isolation. A “low risk patient” (i.e. young and healthy) may still be in the highest risk “Tier 3” based on symptoms, social factors, and/or clinical worsening. Similarly, a “high risk patient” (e.g. age >70 with COPD) can be in the lowest risk “Tier 1” if they are asymptomatic.

1. **Tier 1 (Lowest risk for hospitalization)**:
   1. Definition
      1. No risk factors for severe illness
      2. Unlikely to require medical escalation (telemedicine or in-person)
      3. Main goals for management: (1) application of strict infection prevention measures and (2) clinical monitoring for signs of worsening
      4. Appropriate for scheduled nurse contact by phone every 2 days
   2. Criteria

Asymptomatic patients (any age), **or**

Symptomatic patients who meet all criteria:

- - 1. Patient characteristics (“low risk patient”):
       1. Age <60
       2. No significant comorbidities
    2. Social factors:
       1. Able to self-isolate safely
       2. Adequate caregiver resources in place
    3. Clinical severity:
       1. Upper respiratory symptoms and/or nonspecific symptoms and/or minor lower respiratory tract symptoms (e.g. cough)
       2. No significant mental health symptoms
       3. If recent clinical data available (e.g. ER or UC):
          1. Vital signs normal (exception: fever and tachycardia appropriate to fever are acceptable)
          2. Chest imaging normal
          3. Labs normal (minor liver abnormalities acceptable)
    4. Clinical course:
       1. Stable or improving upper respiratory symptoms and/or nonspecific symptoms and/or minor lower respiratory tract symptoms
       2. If moderate lower respiratory symptoms were previously present, course is improving
       3. Previously Tier 2 patient with improving symptoms over 6 days on follow-up may be recategorized to Tier 1 (decrease risk tier).

1. **Tier 2 (Intermediate risk for hospitalization)**:
   1. Definition
      1. Patient with increased likelihood for medical escalation (telemedicine or in-person) due to presence of minor risk factor(s) for severe COVID-19.
      2. Main goals for management: (1) application of strict infection prevention measures and (2) clinical monitoring for signs of worsening
      3. Appropriate for once daily scheduled nurse contact
   2. Criteria
      1. Patient characteristics (“intermediate risk patient”):
         1. Age
            1. <60 with moderate-risk comorbidity (list below)
            2. 60-69, no comorbidity or mild/controlled comorbidity
         2. Comorbidities:
            1. Diabetes
            2. Hypertension
            3. Asthma
            4. CKD
            5. Tobacco use
            6. Pregnancy (coordinate with OB)

(consider Tier 3 for multiple comorbidities)

- - 1. Social factors:
       1. Able to self-isolate safely
       2. Adequate caregiver resources in place
       3. Otherwise Tier 1 patient with uncertain social factors for isolation/caregiver support can be assigned to Tier 2 (and refer to social resources as available)
    2. Clinical severity:
       1. Upper respiratory symptoms and/or nonspecific symptoms
       2. Minor lower respiratory tract symptoms
       3. Low risk or intermediate risk patient with mild new or worsening depression or anxiety (coordinate with mental health as available)
       4. Otherwise Tier 1 patient, with mild abnormalities in clinical data available (e.g. ER or UC)
          1. Vital signs: resting tachycardia
          2. Low WBC/lymphocytes, elevated CRP
    3. Clinical course:
       1. Stable upper respiratory symptoms and/or nonspecific symptoms
       2. If lower respiratory symptoms present at onset, course is generally improving (mild stable cough acceptable)
       3. Previously Tier 1 tier patient with non-improving symptoms over first 6 days of illness may be recategorized to Tier 2 (increase risk tier).
       4. Previously Tier 3 patient with improving symptoms over 6 days may be recategorized to Tier 2 (decrease risk tier).

1. **Tier 3 (Highest risk for hospitalization)**:
2. Definition
   - 1. Patient with high likelihood for medical escalation due to major risk factor for severe COVID-19: high-risk age or comorbidity, high-risk social factor(s), and/or worsening clinical course
     2. Outpatient-appropriate - does not currently require urgent or emergency evaluation
     3. Main goals for management: (1) close clinical monitoring for signs of worsening including all available data, (2) application of strict infection prevention measures, (3) directing resources to prevent hospitalization for ambulatory-sensitive conditions and social needs, (4) anticipating and facilitating home to hospital transfers
     4. Refer to PCP for complex cases with Goals of Care / Advance Care Planning concern
     5. Appropriate for daily telemedicine visit by provider and daily nurse telephone call (2 check-ins per day)
   1. Criteria
      1. Patient characteristics (“high risk patient”):
         1. Age ≥70
         2. Age <70 with high-risk comorbidity
            1. Cardiovascular disease: CAD or Heart failure, history of stroke
            2. COPD (or other high-risk pulmonary disease)
            3. End stage renal disease
            4. Cirrhosis
            5. Immunosuppression (Biologic or other immunosuppressive medications including chronic corticosteroid at ≥20 mg prednisone daily, detectable HIV VL or CD4 count<200 cells/mm3)
            6. Immunodeficiency
            7. Active malignancy
            8. Frailty (clinical judgement)
            9. Other high-risk medical condition (clinical judgement)
      2. Social factors
         1. Able to self-isolate safely
         2. Adequate caregiver resources in place
         3. If patient is high risk by patient characteristics and has unstable housing and/or caregiver support, consider escalation (or Tier 3 monitoring at provider discretion).
         4. Otherwise Tier 1 patient or Tier 2 patient with unstable housing and/or unstable social support/caregiver resources (coordinate with any social referrals available)
      3. Clinical severity:
         1. High risk patient (by patient characteristics and/or social factors above)
            1. Upper respiratory symptoms and/or mild nonspecific symptoms = Tier 3
            2. Lower respiratory symptoms = Tier 3 vs. escalation, based upon clinical judgement of risk.
         2. Otherwise Tier 2 patient
            1. Minor lower respiratory tract symptoms = Tier 3
            2. Moderate lower respiratory symptoms = Tier 3 vs. escalation, based upon clinical judgement of risk
         3. Otherwise Tier 1 patient with moderate lower respiratory symptoms = Tier 3
         4. Otherwise Tier 1 patient or Tier 2 with other moderate risk clinical data available, subject to clinical judgment:
            1. Chest imaging with mild infiltrates
            2. Mild relative hypoxia (e.g. <4 point change from baseline and >92%; caution: no rapid change in SpO2 or symptoms)
         5. Non-respiratory COVID-19 complication or decompensation in chronic condition amenable to outpatient management (coordinate with PCP/specialty care); e.g. Arrhythmia (consider remote diagnosis as appropriate)
      4. Clinical course
         1. High risk patient (by clinical/social criteria above) with:
            1. Stable or improving upper respiratory symptoms and/or nonspecific symptoms
            2. Stable or improving lower respiratory symptoms
         2. Previously Tier 2 patient with non-improving lower respiratory tract symptoms over 6 days may be recategorized to Tier 3 (increase risk tier).
         3. Previously Tier 1 patient with new moderate lower respiratory symptoms may be recategorized to Tier 3 (increase risk tier); clinical judgment re: further escalation
3. **Clinical symptom/sign definitions**:
   1. Nonspecific symptoms
      1. Fever, chills, malaise, myalgia, anorexia, diarrhea, vomiting, headache
      2. Severe excluded (may require escalation): acute confusion, severe weakness, syncope, acute decline in functional status
   2. Upper respiratory symptoms
      1. Sore throat, congestion, rhinorrhea, sneezing, anosmia
   3. Lower respiratory symptoms
      1. Minor – Cough, sputum production
      2. Moderate – Severe cough, dyspnea on exertion, wheezing or chest tightness with breathing
      3. Severe excluded (escalation):
         1. Highest risk: resting dyspnea, labored breathing, resting pulse oximetry ≤92%.
         2. Other: pleuritic pain, hemoptysis
      4. **A subset of patients with COVID-19 can experience worsening respiratory symptoms and progress quickly to respiratory failure. This can occur shortly after initial presentation or in the second week of illness. A high level of vigilance for worsening respiratory status is needed at each check.**
         1. Suggested questions (cebm.net):
            1. “Is your breathing faster, slower or the same as normal?”
            2. "What could you do yesterday that you can’t do today?”
            3. “What makes you breathless now that didn’t make you breathless yesterday?”
4. **Discharge from our COVID-19 Virtual Management Clinic (not the same as end of mandatory isolation)**
   1. Tier 1
      1. At least **seven** days have passed since symptoms first appeared, **AND**
      2. At least **three** days (72 hours) have passed since resolution of fever without the use of fever-reducing medications, **AND**
      3. Improvement in respiratory symptoms*
   2. Tier 2
      1. At least **fourteen** days have passed since symptoms first appeared **AND**
      2. At least **three** days (72 hours) have passed since resolution of fever without the use of fever-reducing medications, **AND**
      3. Improvement in respiratory symptoms*

- 1. Tier 3
     1. At least **twenty one** days have passed since symptoms first appeared **AND**
     2. At least **three** days (72 hours) have passed since resolution of fever without the use of fever-reducing medications, **AND**
     3. Improvement in respiratory symptoms*

*note: mild lingering cough is permissible if provider has cleared patient from isolation status
