# Supplementary material for: Use of a Telemedicine Risk Assessment Tool to Predict the Risk of Hospitalization of 496 Outpatients With COVID-19: Retrospective Analysis
Source: JMIR Public Health Surveill. 2021 Apr 30;7(4):e25075. doi: 10.2196/25075 (PMC8092025; doi:10.2196/25075)
Supplement: Multimedia Appendix 3 [file publichealth_v7i4e25075_app3.docx]

**Multimedia Appendix 3: Model Building**

1. Assessment of proportionality of hazards assumptom.

a. gender*time interaction is significant

| Covariate | Hazard ratio (59% CI) | p value |
| --- | --- | --- |
| Time*Gender | 0.849 (0.726-0.993) | .041 |
| Gender | 11.136 (1.845-67.229) | .009 |

b. risk Tier*time interaction is not significant

| Covariate | Hazard ratio (59% CI) | p value |
| --- | --- | --- |
| Time*risk tier | 0.965 (0.896-1.038) | .336 |
| Low risk tier | reference |  |
| Intermediate risk tier | 7.92 (1.676-37.412) | .009 |
| High risk tier | 37.739 (4.239-335.985) | .001 |

c. Age*time interaction is not significant

| Covariate | Hazard ratio (59% CI) | p value |
| --- | --- | --- |
| Time*Age≥60 | 0.18 (1.089-0.962) | 1.232 |
| Age≥60 | 0.66 (1.411-0.304) | 6.551 |

d. Reported Obesity*time interaction is not significant

| Covariate | Hazard ratio (59% CI) | p value |
| --- | --- | --- |
| Time*ReportedObesity | 0.92 (0.823-1.028) | .142 |
| ReportedObesity | 6.173 (1.441-26.454) | .014 |

1. Backward stepwise by likelihood ratio and forward stepwise by likelihood ratio

We first start by using a backward stepwise progression by likelihood ratio with the following variables: Tier, Gender, White(2), AgeGTE60, Asthma, Cancer, COPD, CAD, Diabetes, DrugAbuse, HeartFailure, Hypertension, Immunesuppression, LungDisease, ReportedObesity, RenalDisease, SocialSupport .

Tier, AgeGTE60, HeartFailure, Hypertension, and ReportedObesity were in the final model produced by this method.

Forward stepwise progression by likelihood ratio was also performed with the same starting variables and Tier, AgeGTE60, HeartFailure were in the model produced by this methods.

3. Manual assessment of model. With Tier, AgeGTE60, HeartFailure, Hypertension, and ReportedObesity in at least 1 model, variations of these were tried along with strata across gender. Looking at different permutations, only ReportedObesity was significant when added to a base model including Tier, AGEGTE60. HeartFailure and Hypertension were not significant when added to the same model. Likewise adding eiterh HeartFailure or Hypertension to the model containing Tier, AgeGTE60 and ReportedObesity did not lead to any improvement in model fit.

4. Comparison of Risk tier model with Individual covariates

Rstudio used for this as SPSS unable to calculate AIC for Cox models so results are very similar but not exactly like the SPSS output presented in the paper.

a. In assigning risk tier initial symptom severity of lower respiratory symptoms, age, obesity were taken in to account so we started with a model comprised of initial symptom severity, age and obesity and presents of lower respiratory symptoms. The presence of initial symptoms on VOMC intake visit were not significantly related to risk of hospitalization so were removed.

| Covariate | Hazard ratio (95% CI) | p value |
| --- | --- | --- |
| Mild symptom severity | reference |  |
| Moderate symptom severity | 1.8 (0.81-3.99) | .147 |
| Severe symptom severity | 8.98 (1.85-43.49) | .006 |
| Age≥60 | 2.8 (1.38-5.7) | .005 |
| Reported Obesity | 2.69 (1.33-5.48) | .006 |
| Chest Tightness | 0.43 (0.14-1.32) | .139 |
| Wheezing | 1.54 (0.59-4.03) | .378 |
| SOB at rest | 1.84 (0.63-5.39) | .265 |
| SOB with exertion | 1.07 (0.44-2.6) | .88 |
| AIC = 334.8212 |  |  |

b. The approach we took would be analogous of that of mediation by Baron and Kenny with the causal variables being the individual variables and the mediator variable being the risk tier assessment.

Baron, R. M., & Kenny, D. A. (1986). The moderator-mediator variable distinction in social psychological research: Conceptual, strategic and statistical considerations. Journal of Personality and Social Psychology, 51, 1173-1182

Step 1: Show that the causal variables is correlated with the outcome. (X – Y).

The individual covariates/causal variables are significantly related time of hospitalization

| Covariate | Hazard ratio (95% CI) | p value |
| --- | --- | --- |
| Mild symptom severity | reference |  |
| Moderate symptom severity | 1.96 (0.96-4) | .064 |
| Severe symptom severity | 7.79 (2.09-29.12) | .002 |
| Age≥60 | 3.33 (1.68-6.57) | .001 |
| Reported Obesity | 2.77 (1.39-5.52) | .004 |
| AIC = 330.6523 |  |  |

*Gender is included as strata in this model

Step 2: Show that the causal variable is correlated with the mediator. (X-M).

All the independent covariates significantly related to risk tier in linear regression with risk tier as dependent variable and individual covariates as independent variables.

| Covariate | Beta (95%CI) | p value |
| --- | --- | --- |
| Mild symptom severity | reference |  |
| Moderate symptom severity | 0.8 (0.73-0.91) | <.001 |
| Severe symptom severity | 1.4 (1.29-1.75) | <.001 |
| Age≥60 | 0.42 (0.07-0.54) | <.001 |
| Reported Obesity | 0.2 (0.08-0.31) | <.001 |

Step 3: Show that the mediator affects the outcome variable. (M-Y)

Risk tier significantly predicts time to hospitalization

| Covariate | Hazard ratio (95% CI) | p value |
| --- | --- | --- |
| Low risk tier | reference |  |
| Intermediate risk tier | 5.34 (1.54-18.52) | .008 |
| High risk tier | 16.44 (4.8-56.32) | <0.001 |
| AIC = 335.7026 |  |  |

*Gender is included as strata in this model

Step 4: To establish that M completely mediates the X-Y relationship shows that the effect of X on Y controlling for M is zero. If the effect of X on Y controlling for M is still significant but lesser in degree then M is an incomplete mediator.

When both risk tier and symptom severity are both in the model, symptom severity is no longer significant, suggesting that the effects of symptom severity on risk of hospitalization is wholly accounted for by risk tier whereas Age and Obesity are not, suggesting the risk tier model in its current iteration is a complete mediator for symptom severity but an incomplete mediator for Age and Obesity and doesn't fully take into account their effects on risk of hospitalization.

| Covariate | Hazard ratio (95% CI) | p value |
| --- | --- | --- |
| Low risk tier | reference |  |
| Intermediate risk tier | 3.64 (1-13.34) | .051 |
| High risk tier | 10.96 (2.72-44.13) | .001 |
| Mild symptom severity | reference |  |
| Moderate symptom severity | 0.86 (0.38-1.94) | .722 |
| Severe symptom severity | 2.24 (0.53-9.5) | .276 |
| Age≥60 | 2.4 (1.19-4.84) | .015 |
| Reported Obesity | 2.36 (1.18-4.72) | .016 |

*Gender is included as strata in this model

Therefore we kept Age and Obesity in the the final Cox model despite the fact that the AIC are very similar between this model and the original one with individual covariates

| Covariate | Hazard ratio (95% CI) | p value |
| --- | --- | --- |
| Low risk tier | reference |  |
| Intermediate risk tier | 3.75 (1.06-13.31) | .041 |
| High risk tier | 11.01 (3.13-38.73) | <.001 |
| Age≥60 | 2.52 (1.27-5.01) | .008 |
| Reported Obesity | 2.1 (1.06-4.16) | .033 |
| AIC = 328.7987 |  |  |

*Gender is included as strata in this model
