# Supplementary material for: Use of a Telemedicine Risk Assessment Tool to Predict the Risk of Hospitalization of 496 Outpatients With COVID-19: Retrospective Analysis
Source: JMIR Public Health Surveill. 2021 Apr 30;7(4):e25075. doi: 10.2196/25075 (PMC8092025; doi:10.2196/25075)
Supplement: Multimedia Appendix 4 [file publichealth_v7i4e25075_app4.docx]

**Multimedia Appendix 4: Sensitivity Analysis for Obesity**

Actual obesity by BMI (CorrectedObesity) was noted to have a higher unadjusted HR and lower p value than ReportedObesity. Swapping CorrectedObesity into the model with Tier and AgeGTE60 for ReportedObesity led to an adjusted hazard ratio of 3.78 (1.76-8.13, p=0.001) for CorrectedObesity, 2.71 (1.37-5.371, 0.004) for Age≥ 60, 3.66 (1.04-12.87, 0.044) for Tier 2 and 11.26 (3.25-38.99, <0.001) for Tier 3
